# Rapid remission of Hailey-Hailey disease with tirzepatide therapy: A case report

**DOI:** 10.1016/j.jdcr.2026.03.015

**Published:** 2026-03-13

**Authors:** Breethiga Velusamy, Olayemi Sokumbi, Benjamin L. Latimer, Elisha M. Singer

**Affiliations:** a Department of Dermatology, Mayo Clinic, Florida; b Departments of Dermatology and Laboratory Medicine & Pathology, Mayo Clinic, Florida

**Keywords:** Hailey-Hailey disease, glucagon-like peptide-1 receptor agonist, tirzepatide

## Introduction

Hailey-Hailey disease (HHD), also known as benign familial pemphigus, is a rare, autosomal dominant, chronic, intraepidermal blistering genodermatoses characterized by relapsing-remitting painful, pruritic, and malodorous eroded and vegetative plaques. HHD significantly impairs quality of life, and currently, there is no standardized or curative treatment.^1^ Glucagon-like peptide-1 receptor agonists (GLP-1 RA), currently approved for the management of type II diabetes and obesity, are increasingly utilized in dermatological conditions, including psoriasis and hidradenitis suppurativa, due to their anti-inflammatory and metabolic effects.^2^^,^^3^ To our knowledge, we describe the first documented case of complete HHD remission associated with tirzepatide therapy.

## Case report

A male in his 30s presented with a 15-year history of recurrent erythematous, crusted, and weeping plaques on his neck and upper torso, exacerbated by heat and sweating. He had previously received several courses of superpotent topical steroids and oral antibiotics without significant or durable improvement. At present, he noted intense pruritus, crusting, and oozing. He denied systemic symptoms, medication changes, or new exposures. His medical history included prediabetes (glycated hemoglobin 6.0 mmol/mol), obstructive sleep apnea, and obesity (body-mass index of 30.1 kg/m^2^). On the day prior to presentation, his primary care physician initiated tirzepatide 2.5 mg subcutaneously every 7 days for weight management.

**Fig 1:**
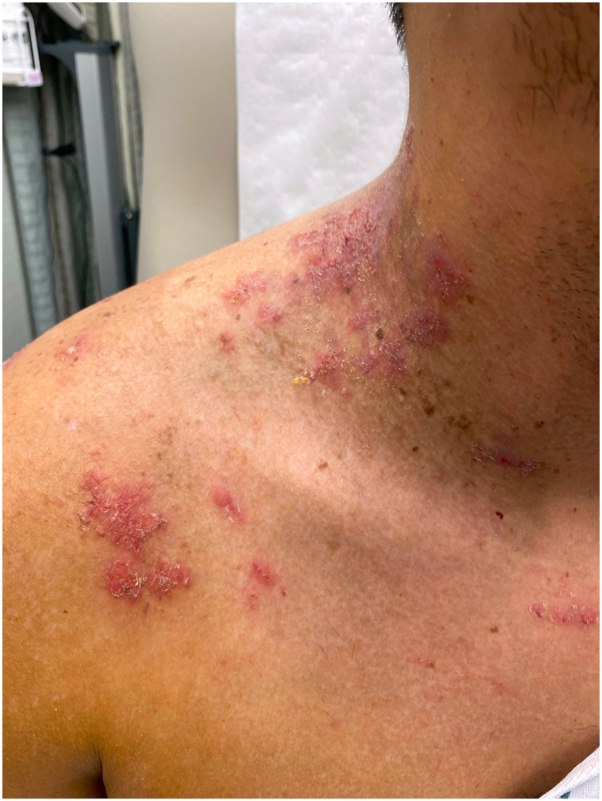
Clinical presentation of Hailey-Hailey disease showing erythematous, crusted plaques on the neck and upper back.

**Fig 2:**
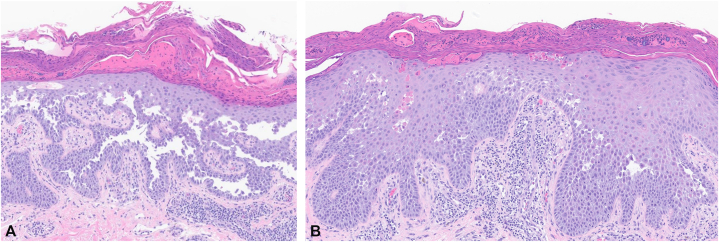
**A,** Histopathological section of the lesion demonstrating diffuse intraepidermal acantholysis with a dilapidated brick wall pattern (*black arrow*) consistent with Hailey-Hailey disease. **B,** Histopathological section of the lesion demonstrating the presence of dyskeratotic cells with eosinophilic cytoplasm in the epidermis (*black arrow*). (**A** and **B,** Hematoxylin-eosin stain; original magnifications: **A,** ×100; **B,** ×15.)

Cutaneous examination of the upper back, shoulders, neck, and anterior torso revealed erythematous, crusted, and eroded plaques (Fig 1). There were no ocular, nasal or oral erosions or ulcerations. An aerobic skin culture grew *Staphylococcus aureus*. A shave biopsy demonstrated diffuse acantholysis with a dilapidated brick wall pattern and dyskeratosis. Based on the clinical presentation of chronic relapsing disease, without mucosal involvement, coupled with the histopathological findings of diffuse acantholytic dyskeratosis, pemphigus vulgaris was ruled out, and a diagnosis of HHD was confirmed (Fig 2).

**Fig 3:**
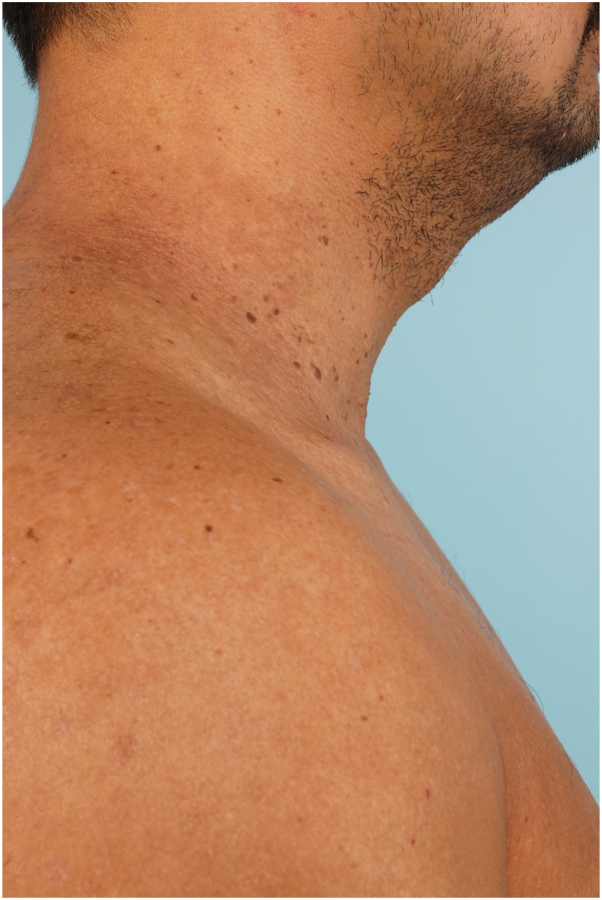
Complete resolution of the lesions at 2-month follow-up after initiation of tirzepatide therapy.

He was prescribed fluocinonide 0.05% ointment and doxycycline 100 mg twice daily, but self-discontinued these after a few days due to lack of improvement. Over the next 2 months, he remained only on tirzepatide therapy, and during this time, he lost 20 pounds of weight. At his follow-up appointment 2 months later, he noted complete resolution of cutaneous symptoms and eruption (Fig 3). Now, 4 months into tirzepatide therapy, he remains in clinical remission. Given that tirzepatide was the only new medication initiated during this period, this strongly suggests that tirzepatide exerted a therapeutic effect on his HHD.

## Discussion

HHD is caused by mutations in the *ATP2C1* gene located in the long arm of chromosome 3 that encodes ATPase 2C1, an adenosine triphosphate (ATP) driven magnesium-dependent calcium pump in the Golgi apparatus. This defect leads to cytosolic accumulation of calcium and alteration of junctional cadherin protein synthesis, resulting in defective cell adhesion termed acantholysis.^4^ Additionally, this mutation reduces mitochondrial ATP production, thereby depleting mitochondrial DNA, which leads to increased reactive oxygen species and decreased keratinocyte proliferation and differentiation, resulting in a fragile epidermis.^5^ In one-third of patients, there is no family history, and these cases are thought to represent those with sporadic de novo mutations.^4^

At present, there are no Food and Drug Administration−approved therapies for HHD, and management is quite challenging. Current treatments are primarily directed toward controlling flares with topical steroids, topical calcineurin inhibitors, oral steroids, and oral antibiotics. Topical cinacalcet, narrow band UV-B, dupilumab, upadacitinib, naltrexone, and naloxone have additionally been reported as therapeutic options. Furthermore, studies show that reductions in sweating with botulinum toxin, oral glycopyrrolate, weight reduction, and avoidance of activities that increase flexural friction may reduce HHD flares.^5^^,^^6^ Despite the current existing treatments, recurrence remains common, underscoring the need for additional disease-modifying systemic therapies.^6^, ^7^, ^8^

Tirzepatide’s mechanism of action in treating HHD may be attributable not only due to overall weight reduction but also to its anti-inflammatory and keratinocyte–migration related effects. GLP-1 receptor signaling in keratinocytes reduces inflammation through at least 2 known immunomodulatory mechanisms. The first is through phosphorylation and activation of the transcription factor cAMP response element-binding protein, which leads to inhibition of the NF-κB inflammasome and reduction of interleukin (IL)-1 signaling and downstream IL-6 production. It is established that downregulation of IL-6 alters ATP2C1 expression and leads to improvements in HHD.^8^ Second, patients with type 2 diabetes and psoriasis treated with GLP-1 RAs displayed significant reductions in dermal gamma delta T cells, resulting in reduced Th-17 mediated inflammation. Recently, IL-17A^+^ cells have been identified within lesional acantholytic and dermal inflammatory HHD skin, suggesting a possible Th17 pathogenic role.^9^ Reviews by Ku and Chang^10^ Krajewski et al,^3^ and Paschou et al^11^ further confirmed that GLP-1 receptor activation downregulates Th17/IL-23 pathways, enhances wound healing, and decreases systemic inflammatory burden. Furthermore, GLP-1 RAs promote keratinocyte migration and wound repair through PI3K/Akt pathways.^12^ Additionally, GLP-1 RAs lead to upregulated adiponectin, which has been shown to influence calcium ATPase in the sarcoplasmic reticulum of cardiomyocytes; a similar mechanism on the calcium ATPase in keratinocytes may be responsible for the improvements noted in HHD.^13^

Barry et al^13^ described remission of refractory HHD with liraglutide therapy. This report, coupled with our patient’s remission on tirzepatide therapy, suggests a potential therapeutic effect of GLP-1 agonists in HHD. Nevertheless, given the small sample size and observational nature of these 2 reports, generalizability is limited, and large scale, prospective, controlled studies are needed to confirm these findings.

## Conclusion

The observed remission of HHD in our patient strongly correlates with the initiation of tirzepatide therapy. While weight loss likely contributed to improvement via reduced friction and sweating, the sustained remission and the mechanistic parallels with prior GLP-1 RA report support a pharmacologic contribution. This case expands on prior observations and suggests that tirzepatide, a dual GLP-1/GIP agonist, may modulate keratinocyte and inflammatory pathways relevant to HHD pathophysiology.

## Conflicts of interest

None disclosed.
